# Mechanisms of HIV non-progression; robust and sustained CD4+ T-cell proliferative responses to p24 antigen correlate with control of viraemia and lack of disease progression after long-term transfusion-acquired HIV-1 infection

**DOI:** 10.1186/1742-4690-5-112

**Published:** 2008-12-11

**Authors:** Wayne B Dyer, John J Zaunders, Fang Fang Yuan, Bin Wang, Jennifer C Learmont, Andrew F Geczy, Nitin K Saksena, Dale A McPhee, Paul R Gorry, John S Sullivan

**Affiliations:** 1 Australian Red Cross Blood Service, 153 Clarence Street, Sydney, NSW 2000, Australia; 2 Transfusion Medicine and Immunogenetics Research Unit, Central Clinical School, Faculty of Medicine, University of Sydney, Sydney, NSW, Australia; 3 Centre for Immunology, St. Vincent's Hospital and University of NSW, Sydney, NSW, Australia; 4 Retroviral Genetics Division, Centre for Virus Research, Westmead Millennium Institute, University of Sydney, Sydney, NSW, Australia; 5 National Serology Reference Laboratory, St Vincent's Institute, Melbourne, VIC, Australia; 6 Department of Microbiology and Immunology, University of Melbourne, Parkville, VIC, Australia; 7 Centre for Virology, Macfarlane Burnet Institute for Medical Research and Public Health, Melbourne, VIC, Australia; 8 Department of Medicine, Monash University, Melbourne, VIC, Australia

## Abstract

**Background:**

Elite non-progressors (plasma viral load <50 copies/ml while antiretroviral naive) constitute a tiny fraction of HIV-infected individuals. After 12 years follow-up of a cohort of 13 long-term non-progressors (LTNP) identified from 135 individuals with transfusion-acquired HIV infection, 5 remained LTNP after 23 to 26 years infection, but only 3 retained elite LTNP status. We examined the mechanisms that differentiated delayed progressors from LTNP in this cohort.

**Results:**

A survival advantage was conferred on 12 of 13 subjects, who had at least one host genetic factor (HLA, chemokine receptor or TLR polymorphisms) or viral attenuating factor (defective *nef*) associated with slow progression. However, antiviral immune responses differentiated the course of disease into and beyond the second decade of infection. A stable p24-specific proliferative response was associated with control of viraemia and retention of non-progressor status, but this p24 response was absent or declined in viraemic subjects. Strong Gag-dominant cytotoxic T lymphocyte (CTL) responses were identified in most LTNP, or Pol dominant-CTL in those with *nef*-defective HIV infection. CTL were associated with control of viraemia when combined with p24 proliferative responses. However, CTL did not prevent late disease progression. Individuals with sustained viral suppression had CTL recognising numerous Gag epitopes, while strong but restricted responses to one or two immunodominant epitopes was effective for some time, but failed to contain viraemia over the course of this study. Viral escape mutants at a HLA B27-restricted Gag-p24 epitope were detected in only 1 of 3 individuals, whereas declining or negative p24 proliferative responses occurred in all 3 concurrent with an increase in viraemia.

**Conclusion:**

Detectable viraemia at study entry was predictive of loss of LTNP status and/or disease progression in 6 of 8, and differentiated slow progressors from elite LTNP who retained potent virological control. Sustained immunological suppression of viraemia was independently associated with preserved p24 proliferative responses, regardless of the strength and breadth of the CTL response. A decline in this protective p24 response preceded or correlated with loss of non-progressor status and/or signs of disease progression.

## Background

A cohort of blood product recipients with transfusion-acquired HIV (TAHIV) infected between 1981 and 1984 was followed prospectively by the Australian Red Cross Blood Service HIV Lookback Team since 1987. There are individuals in this cohort who have remained asymptomatic for 27 years since infection without antiretroviral therapy; some maintaining plasma HIV RNA levels to below detectable levels and a stable CD4 T cell count, thus retaining elite non-progressor status. Early natural history studies on this and other cohorts suggested that TAHIV infection may result in a shorter time to AIDS than sexually-acquired (SA) HIV infection [1,2]. This observed increase in the rate of disease progression in TAHIV may be due to the higher inoculation volume of blood product compared with the much smaller blood or genital fluid exchange involved in SAHIV infection [1], as well as the known immunomodulatory effect of transfusion on immune function [3,4]. Age is also an independent predictor for an increased rate of HIV disease progression [5,6]. The bias toward an aged population requiring transfusion is part of the composite disadvantage of transfusion as a route of HIV infection [1]. In addition to HIV infection, survival may be influenced by the underlying medical cause for transfusion. Yet despite these disadvantages, we previously observed a high frequency of non-progression in this TAHIV cohort after 20 years of infection [7].

Early studies on this cohort of TAHIV patients led to the identification of the Sydney Blood Bank Cohort (SBBC) of long-term survivors [8], and that an attenuated *nef*-deleted strain of HIV-1, transmitted from a single donor resulted in slow to non-progression in these individuals [9]. However, after prolonged infection, not all SBBC members maintained non-progressive disease [10-13]. Although HLA type did not explain non-progression in this group [14], we have observed differences in CD8 T cell responses that are associated with HLA-dependent epitope recognition [15], and we have detected increased preservation of helper T cell responses in non-progressors from this cohort [16,17]. In addition to the well described host genetic factors which may prolong non-progression [7], recent studies have suggested an influence from innate immune mechanisms, including polymorphisms that decrease TLR function thereby reducing immune activation upon exposure to infections diseases [18], or the FcγRIIA polymorphism (R/R) which is strongly associated with progressive HIV disease as a result of impaired elimination of HIV immune complexes [19].

While host genetic factors may predispose an individual for delayed disease progression, there is substantial evidence that antiviral T cell responses are required to sustain non-progressor status. Earlier studies have demonstrated an important role for Gag-specific CTL in delaying disease progression [20,21]. Non-progressors that control viraemia in the absence of antiviral therapy also have strong CD4 T cell proliferative responses to the Gag protein p24 [22]. Importantly, for Gag CTL to be efficient in killing HIV-infected cells and therefore protective in controlling viraemia, these must also be accompanied by p24-specific T cell proliferative responses [23-25]. Appropriate T cell help is also required to achieve maturation and display of effector phenotypes on CTL associated with effective virological control [26].

To determine how these host genetic and immune factors combined to contribute to prolonged non-progression in our TAHIV cohort, we report here on the current status of the elite non-progressors not on antiretroviral therapy (ART), examining the factors that have influenced disease in the former non-progressors (now on therapy or deceased), and analyse potential mechanisms that have influenced non-progression in this cohort for up to 27 years.

## Materials and methods

### Definitions of non-progression and disease progression

When this prospective study began in 1994, 13 LTNP were identified in the NSW TAHIV cohort according to the original guidelines for classifying LTNP: at least 10 years infection, stable CD4 T cell counts >500 cells/μl, and no history of ART [27,28]. Subsequently, loss of LTNP status was defined by any of the following events: a consistent decline in CD4 T cell counts below 500/μl, commencement of ART, and after viral load testing became routine, plasma viraemia >5000 copies/ml. Elite non-progressors were also defined by viraemia suppressed to <50 copies/ml in addition to the above criteria. Disease progression was defined by a CD4 T cell count of <200 and/or plasma viraemia >100,000 copies/ml.

### Patient details

The two non-progressor groups in this study included the SBBC, consisting of 6 recipients of HIV-infected blood from a common donor, and the other (Cohort 2) consisting of 7 recipients infected by blood from different donors. Clinical data from these LTNP were collected prospectively since the late 1980s. T cell counts and viral load tests were performed as part of routine clinical care. Blood samples and clinical histories were provided after informed consent was granted in accordance with guidelines from the ARCBS institutional Human Research Ethics Committee.

### T cell functional analyses

Anti-HIV T cell function assays were performed as previously described [15,29]. Briefly, the proliferative response to HIV-1 p24 was determined by 6 day culture of PBMC (1 × 10^5 ^cells/well) in RPMI medium with 15% pooled human serum in round bottom microtitre plates, with 2 μg/ml HIV-1_SF2 _p24 (Chiron, Emeryville, CA, USA), or medium alone for control. After 6 days, proliferative responses were determined by ^3^H-thymidine incorporation during a further 6 hours culture, followed by cell harvest and reading in a liquid scintillation counter. Results were expressed as stimulation index (SI; mean counts antigen wells/mean counts control wells), and a SI >3 was considered a positive response.

The response of CD8+ T cells to HIV antigen was measured by IFNγ ELISPOT, using pre-coated ELISPOT kits according to the manufacture's protocol (Mabtech, Mosman, Australia). Firstly, the response to whole HIV proteins was determined in response to antigen presented by autologous B lymphoblastoid cell lines infected for 18 hours with 5 pfu/cell recombinant Vaccinia expressing the HIV-1_IIIB _*env*, *gag*, *pol*, or *nef *genes (Therion Biologics, Cambridge, MA, USA), or E. coli *lacZ *as a control. Gag responses were further characterised using overlapping Gag peptides, firstly using a matrix of peptide pools, and then individual peptides for confirmation (full Gag peptide set; kindly provided by the NIH AIDS Research and Reference Reagent Program, Division of AIDS, NIAID, NIH).

### Provirus sequencing

DNA from PBMC was isolated using a QIAamp DNA mini kit (Qiagen, Valencia, CA) according to the manufacturer's protocol. A nested polymerase chain reaction (PCR) was used to amplify ~1.5 kb of the HIV gag gene using the following primers:

5'-TCTCGACGCAGGACTCGGCTTGCTGA-3' (outer, sense),

5'-TACTGTATCATCTGCTCCTGTAT-3' (outer, antisense),

5_-GACAAGGAACTGTATCCTTTAGCTTC-3 (inner, sense),

And 5'-TCTGCTCCTGTATCTAATAGAGCTT-3' (inner, antisense).

Both primary and secondary PCR reactions contained 2 units of Taq DNA polymerase (Promega, Madison, WI), 1× PCR buffer (Promega: 1 mM Tris- HCl, 5 mM KCl, 0.1% Triton X-100), 2.5 mM MgCl2, 200 nM of each dNTP, and 0.4 nM of each primer in a total volume of 50 ul. Thermocycling conditions were as follows: 95°C for 2 min and then 35 cycles of 94°C for 30 s, 55°C for 30 s, 72°C for 2 min and a final a single cycle of 72°C for 7 min.

RNA was isolated from plasma using the QIAamp Viral RNA Mini Kit (Qiagen, Valencia, CA) according to the manufacturer's protocol. Gag gene was amplified using the QIAGEN OneStep RT-PCR Kit using the outer primer pairs mentioned above. Second round PCR reactions were performed using the inner primer pair under the same conditions.

PCR products were purified using a Millipore PCR purification plate (Millipore, Billerica, MA, USA) and sequenced by the ABI PRISM BigDye Terminator V3.1 Ready Reaction Cycle Sequencing kit (Applied Biosystems, Foster City, CA, USA) on an ABI 377 automated sequencer. Multiple sequences derived from each patient were analysed using Sequencher 3.11 software (Gene Codes Corp., Ann Arbor, MI, USA). Chromatograms derived from both forward and reverse primers were aligned with the reference strain HIV-1 HXB2.

### Host genetic typing

Methods for HLA and chemokine receptor polymorphisms [30] and toll-like receptor (TLR) and FcγRIIA polymorphisms [31-33] have been described elsewhere.

### Statistical analysis

The Fishers Exact test was used to associate genetic and immune factors with viraemia and non-progressor status.

## Results

### Status of the non-progressor cohort

From all reported TAHIV cases from the state of NSW, Australia, a cohort of 13 (10%) remained asymptomatic after 10 years of infection. We now report that only 5 remain non-progressors after 23 to 26 years of HIV-1 infection. Infection and treatment history for each subject is summarised in Additional file 1. Most of these individuals had a survival advantage, with 7 of 13 having at least one host genetic polymorphism associated with slow progression, and 6 of 13 were infected with the SBBC *nef*-defective HIV-1 strain [12], and combined, 12 of 13 had at least one host or viral factor favouring slow progression. Acting in opposition to these survival advantages, 5 of 8 former non-progressors had the FcγRIIA polymorphism (R/R). While this genotype was absent in current LTNP, the effect of the R/R genotype in promoting disease progression was not significant in this small study of 13 individuals. On balance, these competing survival factors along with antiviral immune responses enabled a non-progressive disease course to be established early in infection.

The loss of non-progressor status was based on increasing viraemia and/or decreasing CD4 counts in 5 of 8, and initiation of ART in these individuals (Additional file 1). Patient C122 lost LTNP status due to gradually increasing viraemia, but died from unrelated causes before substantial T cell loss was observed. Another two elderly individuals (C18 and C54; both SBBC members), each with low detectable viraemia, died before losing their non-progressor status [13,17].

### Antiviral immune responses associated with non-progression

**Figure 1:**
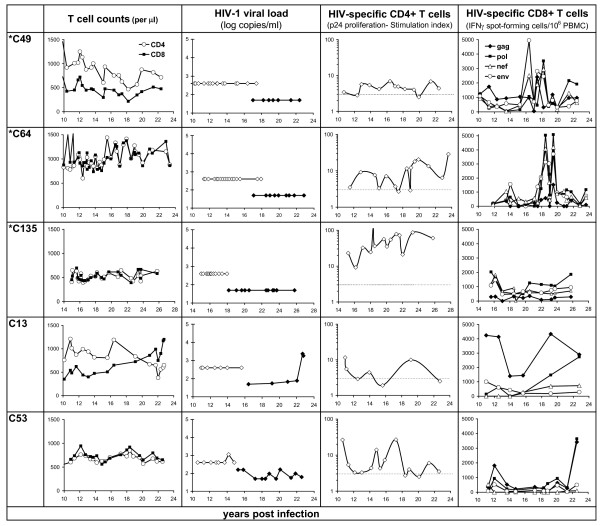
Immunovirological status of the surviving non-progressors, showing T cell counts; viral RNA copies/ml plasma (data generated from the Roche Amplicore standard assay, limit of detection 400, and Ultrasensitive assay, limit of detection 50, plotted separately); T cell proliferative responses to recombinant HIV-1 p24 (stimulation index; significant responses >3, defined by the broken line); and IFNγ responses (ELISPOT) by CTL against autologous BCL expressing HIV-1 antigens after infection with recombinant vaccinia. *SBBC member.

**Figure 2:**
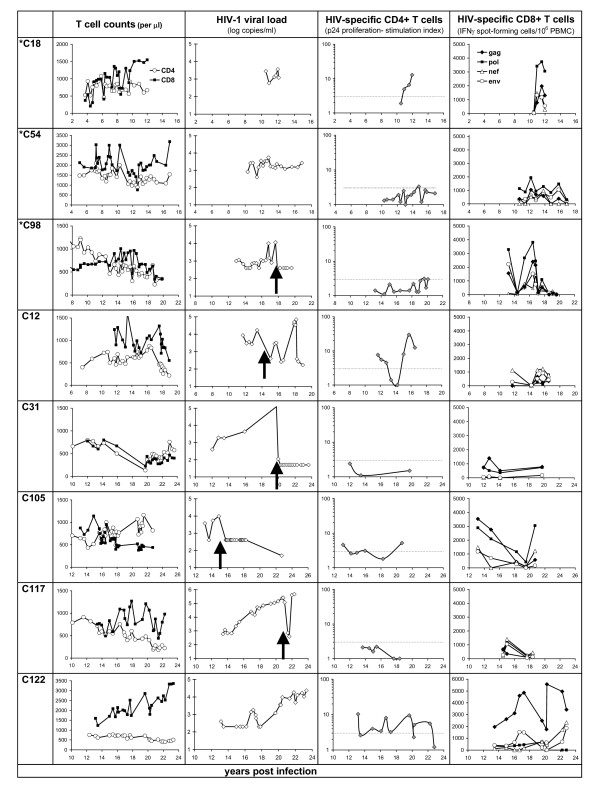
**Immunovirological status of the former non-progressors **(same parameters as in figure 1). Initiation of antiretroviral therapy is defined by an arrow in the viral load panels. Other reasons for loss of non-progressor status are summarised in Additional file 1.

Host and viral genetic factors may have played a role in delaying disease progression into the second decade of infection in these 13 individuals, but this study also demonstrates the importance of host immune responses in sustaining this non-progressive disease course into and beyond the second decade of infection. Immune status and activity of HIV-specific CD4 T cells (proliferation) and CD8 T cells (IFN-γ response) is shown for the current non-progressors (Figure 1) compared with those that lost their non-progressor status or died (Figure 2).

Antiviral CTL responses were variable during the second decade of HIV infection, and did not always correlate with viremia for members of these cohorts. Strong Gag-specific CTL were detected in the Cohort 2 non-progressors (C13, C53, C122, and C105 before ART), but the predominant CTL response in the SBBC members was against Pol antigens. These CTL appeared to be equally effective in containing viral replication, whether Gag-specific as demonstrated in earlier time points in C122, or Pol-specific in C18 (Figure 2).

The main factor that differentiated LTNP from those that lost non-progressor status, was low or undetectable HIV viraemia (<100 copies/ml; p = 0.021), and low viraemia was associated with detectable p24 proliferative responses (p = 0.0047). Loss of non-progressor status was strongly associated with undetectable or declining p24 responses (p = 0.0047). The combination of detectable p24 proliferative responses and strong (>500 SFC/10^6 ^PBMC) Gag CTL responses was associated with low (<100 copies/ml) or undetectable viraemia (p = 0.032).

**Figure 3:**
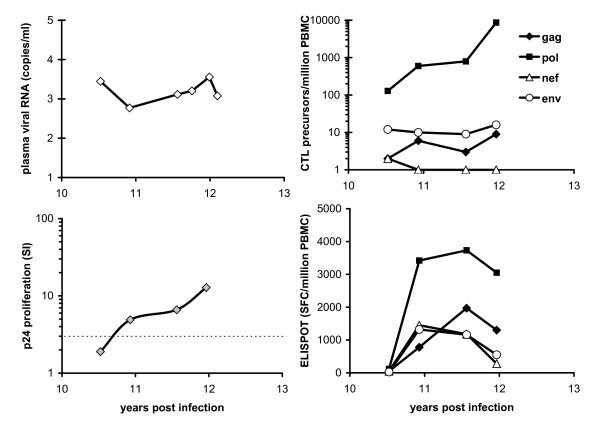
Dynamics of immune responses during an episode of increased viral replication in SBBC patient C18.

Illustrating the importance of these combined Gag-specific T cell responses over time, low viraemia was intermittently detected at earlier time points in C122, with sharp increases in Gag CTL temporally associated with control of transient viraemia at 17 years post infection. However, Gag CTL later failed to contain viraemia in C122 beyond approximately 20 years, coinciding with weakening proliferative responses that gradually became negative. A similar correlation between anti viral immune responses and a spike in viral replication was demonstrated in SBBC member C18, shown in more detail in Figure 3. Over the course of 12 months, in response to an increase in viraemia peaking at 3600 copies/ml, the p24 proliferative response increased, along with substantial expansions of Pol-specific CTL in both precursor [15] and effector CTL populations. The durability of immune control in this individual was not determined as he died soon after from causes unrelated to HIV disease, aged 83.

A decline in Gag-specific T cell responses preceding detectable viraemia was demonstrated in C13. This decline up to year 16 was followed by a period of low detectable viraemia (50 – 100 copies/ml) between years 19 – 22. A rebound in these Gag-specific T cell responses coincided with the first detectable viraemia at 19 years. These T cell responses may have helped contain viraemia to low levels over the following two years, but the sharp increase in viral RNA at 22.7 years (Figure 1) coincided with a decline in Gag-specific CD4 and CD8 T cell responses, whereas Pol-specific CTL increased in response to rising viraemia. These examples demonstrate the influence of conserved Gag-specific responses, particularly helper T cell responses, in reduced viral replication and delayed disease progression. While the decline in these responses preceded detectable viraemia in C13, sufficient patient specimens were not available to allow this critical observation to be made in others who progressed.

### Breadth of the anti-Gag CTL response in non-progressors

**Figure 4:**
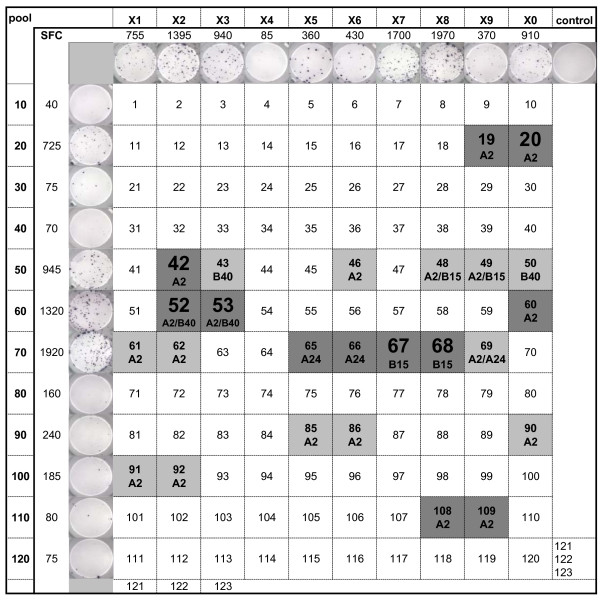
**Identification of responses to Gag peptide epitopes by peptide pool mapping in a stable non-progressor (C53, 21.3 years post infection)**. Mean INF-γ spots/10^6 ^PBMC (SFC), and representative ELISPOT images are shown. Individual peptides intersecting positive peptide pools containing HLA-relevant epitopes (Additional file 2) were then tested individually, and positive responses indicated by dark shaded cells, and dominant responses in large font.

**Figure 5:**
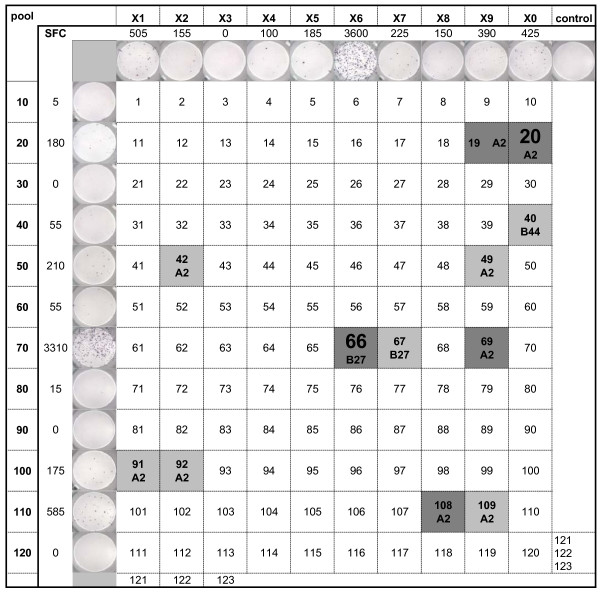
**Identification of responses to Gag peptide epitopes by peptide pool mapping in an individual with increasing viraemia (C122, 20.3 years post infection)**. Mean INF-γ spots/10^6 ^PBMC (SFC), and representative ELISPOT images are shown. Individual peptides intersecting positive peptide pools containing HLA-relevant epitopes (Additional file 2) were then tested individually, and positive responses indicated by dark shaded cells, and dominant responses in large font.

**Figure 6:**
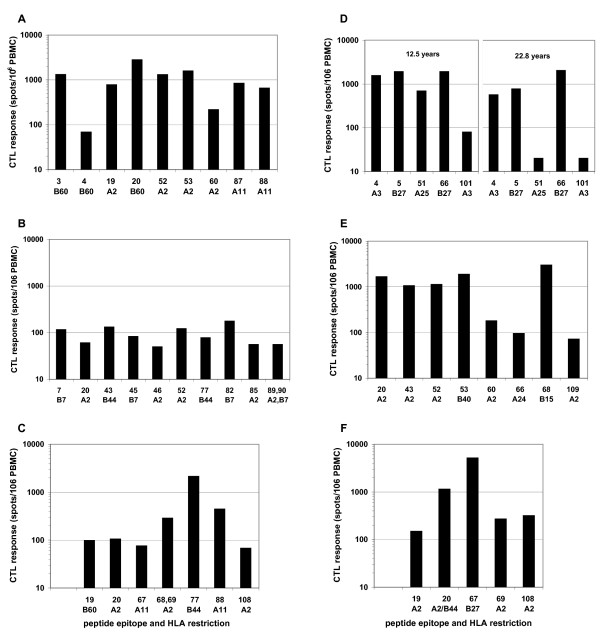
Breadth of Gag CTLs, showing responses to individual peptides selected from intersecting positive peptide pools, in non-progressor C49 (A), C64 (B), C18 (C), C13 showing an early and late time point (D), C53 (E), and C122 (F). Limit of detection 50 spots/10^6 ^PBMC.

To determine why strong Gag CTL may have contained viral replication in some, but failed in others, we mapped the breadth of the Gag CTL response over time in patients with at least moderate CLT responses to whole Gag antigens. Pools of overlapping 15-mer Gag peptides were used to test sequential PBMC spanning the study period by ELISPOT. The composition of each peptide pool, and examples of responses to these are shown in Figures 4 and 5, indicating the relevant HLA-specific epitopes contained in peptides at the intersection of positive pools. Figure 4 demonstrates a broad strong response by C53's PBMC to multiple immunodominant epitopes, contrasted in Figure 5 by the restricted response from C122 to only two immunodominant epitopes. The sequential analysis revealed relatively high stability in the repertoire of Gag responses over the past 10 years in most subjects (Additional file 2). Relevant epitopes at intersecting positive peptide pools were then confirmed using individual peptides (Figure 6). This data demonstrates that retention of broadly reactive Gag CTL was associated with ongoing non-progression (C49, C64, and C53), while restriction toward a narrow CTL specificity was observed in patients that eventually lost control of viraemia (C122 and possibly C13). The SBBC non-progressors C49 and C64 had responses to several Gag epitopes, and although Gag responses were moderate to weak in C64, this needs to be viewed in the context of Pol CTL dominance in the SBBC. A strong but restricted Gag response was also seen in C18, but these Gag responses were likely to be secondary in controlling viraemia, as suggested by the kinetics of Pol CTL in response to a spike in viraemia (Figure 3). Pol CTL recognition was confirmed by subsequent analysis of responses to peptide pools derived from the full set of Pol overlapping 15-mer peptides. Moderate to strong responses to multiple pools containing epitopes in the reverse transcriptase protein were detected in SBBC members C49, C64, C18, C54, but weakly in C98 (data not shown). C18 also responded strongly to integrase peptides.

A strong but narrow CTL response may eventually fail to control viral replication. Restricted recognition of only one A3 and two B27 Gag epitopes in C13 appeared sufficient to have contained viraemia for many years, but the most recent viral load result (Figure 1) suggested that immune escape from these B27-restricted CTL may have occurred recently. Similarly, the predominant response by C122 against an immunodominant B27 epitope (Figure 5 and 6) may have contained earlier spikes of increased viraemia, but ultimately failed to contain increasing viral replication in later years (Figure 2).

### Limited immune escape from HLA B27-restricted CTL

**Figure 7:**
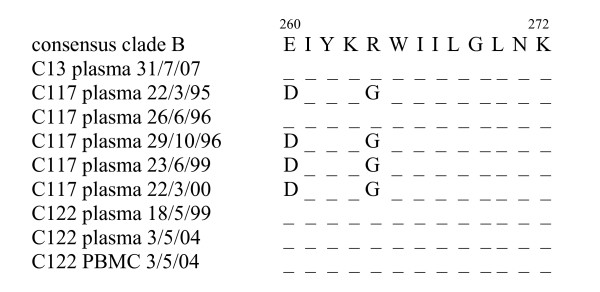
Amino acid sequences of the HLA B27 restricted Gag epitope KRWIILGLNK).

To determine why immunodominant B27-restricted CTL initially contributed to reduced viral replication in C13 and C122, but not in C117, sequencing of plasma and PBMC derived virus spanning the period before and after signs of disease progression was carried out to determine if viral escape mutants had emerged in this region of Gag (Figure 7). With the exception of one sample in 1996, a well characterised escape mutant [34] was detected from the earliest time point in C117. This escape mutant was not detected in C13 or C122, and hence was not the cause for the loss of control of viraemia in C122, nor was it detected in the latest time point from C13 when viraemia first increased above 1000 copies/ml. This suggests that immune escape at this B27 Gag epitope was not a major cause of disease progression in very long term infected individuals. The sole common factor was a decline in p24-specific proliferative responses.

## Discussion

Non-progressors are considered to represent the tail end of the distribution curve of rates of disease progression, and although elite non-progressors are extending this curve even further, disease progression may be inevitable in this rare group of individuals. Recent analyses of the SBBC may support this suggestion [13,17]. However, death from other causes has prevented the establishment of definitive proof of disease progression in some individuals. Two SBBC subjects that did not consent to prospective analysis died from unrelated causes in 1987 and 1994, and the sole SBBC recipient on therapy (C98) has since died from non-HIV causes. Two other elderly subjects also died from non-HIV causes (C18 and C54), but control of viraemia at low levels along with normal CD4 T cell counts suggested there was no evidence for loss LTNP status before death. This leaves the three elite non-progressors from the SBBC described in this study, and one is also advanced in age. One of the elderly Cohort 2 LTNP (with wild-type HIV infection) also died recently from non-HIV causes aged 84 (C122). We may have an opportunity to determine the factors involved in disease progression in the other two Cohort 2 non-progressors (C13 and C53). Both had very low but detectable viraemia, but a recent inversion of CD4 : CD8 T cell ratio in C13 is evident of a change in HIV-induced immune activation. Based on the decline in the proliferative response to p24 preceding this recent increase in viraemia, it is likely that these together signify a transitionary stage toward disease progression in C13.

The p24 proliferative response was the single immune parameter that consistently defined control of viraemia and non-progression. The p24 proliferative response may also be specifically protective, as suggested by a study showing that responses to some Pol antigens were associated with increased viraemia, whereas a protective association was always found in responses to Gag antigens [35]. HIV-specific proliferative responses may also promote CTL proliferation. This was supported by the response to a spike in viraemia in patient C18, where the HIV-specific CD4 and CD8 memory T cell pool increased and were maintained throughout this period (both proliferation and CTL precursor assays measure proliferating antigen-specific memory T cells), whereas effector CTL (ELISPOT-positive cells) peaked then declined as viraemia declined. These data suggest that effective helper T cell function involves proliferation followed by maturation into both effector and costimulatory cells that provide "help" for other lymphocyte functions. Thus, antigen-specific CD8 T cell proliferation may be directly associated with CD4 proliferation to epitopes on the corresponding antigen. It is also possible that loss of protective CD4 and CD8 HIV-specific proliferation may be mediated by a common immunological defect.

The other distinguishing feature of most slow and non-progressor subjects that we have studied is the predominance of Gag CTL, but the SBBC non-progressors were exceptional in having a Pol-dominant CTL response. This observation is unexpected, considering the extensively published role for Gag CTL in controlling viraemia [20,21,23]. Also peculiar to SBBC members, the strongest CTL responses were detected in those with detectable viraemia, and were weaker in subjects with undetectable viraemia [15]. In the non-attenuated HIV-infected non-progressors, strong immunodominant CTL combined with detectable proliferative responses to p24 appears to have contributed to viraemia remaining <100 copies/ml after 23 years HIV-1 infection in patient C53. The individual with the strongest CTL response was C122, but CTL increased as viraemia increased in this patient, while proliferative responses to Gag p24 declined. Given the predominant CTL response in this subject was directed against an immunodominant HLA B27 restricted p24 epitope, and that there were no immune escape sequences detected at this epitope, it is likely that the decline in the p24-specific proliferative response was the key event that contributed to the failure of CTL to control viraemia, as it is understood that CTL have much reduced functional efficiency in containing viraemia in the absence of helper T cell responses [24]. Another study of HLA B57 positive individuals found no association between disease progression and the strength of CTL responses or the emergence of viral escape mutants at these epitopes, but it was found that viral replicative fitness influenced disease course [36]. The contribution of p24-specific proliferative responses was not investigated in that study.

The neutralising antibody (NAb) response is another immune mechanism that may contribute to long term control of viraemia [37]. We recently analysed viral replicative fitness and the strength of NAb responses, and confirmed that NAb titres in long-term infected subjects were inversely proportional to viral load. However, NAb titres in SBBC members were comparatively weaker, and in parallel with CTL responses, were highest in those with detectable viraemia. The presence of strong Nabs did not prevent some SBBC members from developing signs of disease progression [38]. Hence, we suggest that while broad Nabs might be generated due to a crippled infection they do not prevent disease progression, particularly in the absence of antiviral helper T cell responses.

What is the key factor that sustains a non-progressive disease course, and what initiates the decline in protective immunity after many years of a non-progressive disease course. Could a change in viral pathogenicity overcome this delicate balance between host and virus, or could progressive weakening of the CD4 T cell response by slow virus turnover gradually allow the virus to escape the combined effector mechanisms of the HIV-specific cell-mediated and humoral immune responses? Escape mutants at the B27 epitope KRWIILGLNK have been observed under conditions of high viral load and evolutionary drift [34], which was likely in C117, but not for both C13 and C122, who had prolonged periods of immune control in the presence of p24 proliferative responses, and very low viraemia up to or beyond the second decade of HIV infection. Disease progression in one SBBC member (C98) and the SBBC infecting donor was associated with the emergence of divergent strains which preceded viral load increases and subsequent changes in immune responses [39,40]. Also, these individuals lacked protective p24 proliferative responses and had detectable viraemia before viral divergence occurred, providing further evidence that effective immune control over viral replication to levels below a putative threshold, may prevent the emergence of escape mutants or fitter variants. Therefore, the common factor in all these observations is the decline or lack of p24 proliferative responses, suggesting that a lack of helper T cell responses may result in a reduced capacity to contain viral replication by other immune effector responses including CTL, independent of the presence of viral escape mutants.

## Conclusion

Our studies have demonstrated that host and viral genetic factors can contribute to delayed disease progression, but the single immunological factor that functionally defined non-progression was Gag-specific CD4 T cell proliferation. The maintenance of this p24-specific response does not require detectable viral replication for antigenic stimulation [41]. Detectable p24-specific T cell proliferation defines the immunocompetent recall response to viral antigen, and when spikes of viral replication were detected in these individuals, these cells likely provided T cell help for maintaining functional antiviral effector responses by other CD4 and CD8 T cells, including non-cytolytic antiviral mechanisms [41], and may also provide T cell help for efficient generation of NAb by HIV-specific B cells. We have demonstrated that a decline in this protective p24 response in slow progressors either preceded or coincided with classic signs of disease progression.

## Competing interests

The authors declare that they have no competing interests.

## Authors' contributions

WBD wrote the manuscript and directed the immunology experimental work. BW and NKS provided the sequence data, and JCL provided patient clinical details. FFY provided host genetic data. JJZ and ADK assisted with analysis of T cell immunity data, and DM and PRG provided commentary on neutralising antibody and viral evolution and helped edit the manuscript. AFG and JSS contributed to the design of the LTNP studies.

## Supplementary Material

Additional File 1 Clinical status of the study subjects. ^¶^LTNP cohort identified in 1994, consisting of the SBBC and "cohort 2" non-progressors. ^‡^SBBC members. * Data current at 1/1/2008, or at time of death. ^§^Antiretroviral therapy (date commenced). ^Δ^Genotypes associated with slow disease progression are indicated in bold font, increased disease progression by bold italic font. TLR polymorphisms: TLR2 753 Arg/Gly; TLR4 299 Asp/Gly; TLR4 399 Thr/Ile. wt (wild type, or default genotype). Click here for file

Additional File 2 Sequential T cell reactivity (indicated by X) detected by INF-γ ELISPOT against HIV-1 Gag T cell epitopes (identified by responses at intersecting peptide pools). Sequential T cell reactivity (indicated by X) detected by INF-γ ELISPOT against HIV-1 Gag T cell epitopes (identified by responses at intersecting peptide pools) throughout the study period in living non-progressors (A) C49, (B) C64, (C) C13, (D) C53, (E) in a deceased SBBC non-progressor with low viraemia (C18), and (F) in a deceased Cohort 2 non-progressor who lost viral control (C122). Individual peptides were tested on PBMC from at least one time point per recipient, and the magnitude of responses reported as spot forming cells/10^6 ^PBMC, with limit of detection at 50 (<). Click here for file
