# Supplementary material for: Mechanisms of HIV non-progression; robust and sustained CD4+ T-cell proliferative responses to p24 antigen correlate with control of viraemia and lack of disease progression after long-term transfusion-acquired HIV-1 infection
Source: Retrovirology. 2008 Dec 11;5:112. doi: 10.1186/1742-4690-5-112 (PMC2633348; doi:10.1186/1742-4690-5-112)
Supplement: Additional File 1 — Clinical status of the study subjects. ¶LTNP cohort identified in 1994, consisting of the SBBC and "cohort 2" non-progressors. ‡SBBC members. * Data current at 1/1/2008, or at time of death. §Antiretroviral therapy (date commenced). ΔGenotypes associated with slow disease progression are indicated in bold font, increased disease progression by bold italic font. TLR polymorphisms: TLR2 753 Arg/Gly; TLR4 299 Asp/Gly; TLR4 399 Thr/Ile. wt (wild type, or default genotype). [file 1742-4690-5-112-S1.doc]

| study subject | DOB | age at infection | date  T’fused | years*  HIV+ | age* | ART ^§^  status | ^Δ^ viral and host genetic factors associated with: | | | | | | reasons for  loss of LTNP status |
| --- | --- | --- | --- | --- | --- | --- | --- | --- | --- | --- | --- | --- | --- |
|  |  |  |  |  |  |  | slow disease progression . | | | | | progression |  |
|  |  |  |  |  |  |  | Δnef | CCR2 | CCR5 | HLA | TLR | FcγRIIA |  |
| current  non-progressors: |  |  |  |  |  |  |  |  |  |  |  |  |  |
| C49^‡^ | 9/6/1954 | 30 | 11/6/1984 | 23.6 | 53 | naive | **Δnef** | wt / wt | wt / wt | A 2,11  B 7,60 | wt | H/H |  |
| C64^‡^ | 20/3/1926 | 57 | 4/5/1983 | 24.7 | 81 | naive | **Δnef** | wt / wt | wt / wt | A 2,32  B 7,44 | wt | R/H |  |
| C135^‡^ | 23/2/1946 | 35 | 20/2/1981 | 26.9 | 61 | naive | **Δnef** | wt / wt | **Δ32/wt** | A 1,33  B 50,**57** | wt | R/H |  |
| C13 | 20/5/1946 | 38 | 28/10/1984 | 23.2 | 61 | naive | wt | wt / wt | wt / wt | A 3,25  B 18,**27** | **TLR4**  **299/399** | R/H |  |
| C53 | 5/6/1947 | 37 | 2/8/1984 | 23.4 | 60 | naive | wt | wt / wt | wt / wt | A 2,24  B 15,40 | not  done | not  done |  |
| former  non-progressors: |  |  |  |  |  |  |  |  |  |  |  |  |  |
| C18^‡^ | 12/12/1912 | 70 | 31/8/1983 | 12.2 | dead  (83) | naive | **Δnef** | ND | wt / wt | A 2,11  B 44,60 | **TLR4**  **299/399** | ***R/R*** | not applicable  (non-AIDS death) |
| C54^‡^ | 17/2/1928 | 55 | 24/7/1984 | 17.1 | dead  (72) | naive | **Δnef** | wt / wt | wt / wt | A 25,32  B 18,35 | wt | R/H | not applicable  (non-AIDS death) |
| C98^‡^ | 11/7/1937 | 44 | 1/1/1982 | 20.3 | dead  (64) | 17/9/99 | **Δnef** | wt / wt | wt / wt | A 2,28  B 7,60 | wt | ***R/R*** | Progression and ART (non-AIDS death) |
| C12 | 2/4/1951 | 32 | 23/9/1983 | 20.7 | dead  (53) | 11/2/98 | wt | **64I / wt** | **Δ32/wt** | A 11,23  B 44,55 | wt | ***R/R*** | Progression and ART (non-AIDS death) |
| C31 | 29/11/1948 | 34 | 28/9/1983 | 24.3 | 59 | 25/6/03 | wt | wt / wt | wt / wt | A 2,24  B 15,**57** | wt | H/H | progression  and ART |
| C105 | 4/1/1957 | 25 | 3/1/1982 | 26.0 | 51 | 1/11/96 | wt | wt / wt | wt / wt | A 1,31  B 42,56 | **TLR2**  **753** | ***R/R*** | viraemia  and ART |
| C117 | 21/11/1922 | 59 | 2/11/1981 | 24.3 | dead  (83) | 1/12/02 | wt | **64I / wt** | **Δ32/wt** | A 3,66  B 7,**27** | wt | ***R/R*** | progression and ART (non-AIDS death) |
| C122 | 14/12/1919 | 62 | 9/2/1982 | 23.3 | dead  (85) | naive | wt | wt / wt | wt / wt | A 2,31  B **27**,44 | wt | R/H | increasing viraemia  (non-AIDS death) |
