# Supplementary material for: Mechanisms of HIV non-progression; robust and sustained CD4+ T-cell proliferative responses to p24 antigen correlate with control of viraemia and lack of disease progression after long-term transfusion-acquired HIV-1 infection
Source: Retrovirology. 2008 Dec 11;5:112. doi: 10.1186/1742-4690-5-112 (PMC2633348; doi:10.1186/1742-4690-5-112)
Supplement: Additional File 2 — Sequential T cell reactivity (indicated by X) detected by INF-γ ELISPOT against HIV-1 Gag T cell epitopes (identified by responses at intersecting peptide pools). Sequential T cell reactivity (indicated by X) detected by INF-γ ELISPOT against HIV-1 Gag T cell epitopes (identified by responses at intersecting peptide pools) throughout the study period in living non-progressors (A) C49, (B) C64, (C) C13, (D) C53, (E) in a deceased SBBC non-progressor with low viraemia (C18), and (F) in a deceased Cohort 2 non-progressor who lost viral control (C122). Individual peptides were tested on PBMC from at least one time point per recipient, and the magnitude of responses reported as spot forming cells/106 PBMC, with limit of detection at 50 (<). [file 1742-4690-5-112-S2.doc]

Sequential T cell reactivity (indicated by X) detected by INF-γ ELISPOT against HIV-1 Gag T cell epitopes (identified by responses at intersecting peptide pools) throughout the study period in living non-progressors (A) C49, (B) C64, (C) C13, (D) C53, (E) in a deceased SBBC non-progressor with low viraemia (C18), and (F) in a deceased Cohort 2 non-progressor who lost viral control (C122). Individual peptides were tested on PBMC from at least one time point per recipient, and the magnitude of responses reported as spot forming cells/10^6^ PBMC, with limit of detection at 50 (<).

A

| C49 | HLA specificity | reactivity over sequential time points  (years post infection) | | | | |
| --- | --- | --- | --- | --- | --- | --- |
| peptide(s) | A2, 11  B7, 60 | 11.3 | | 17.4 | 19.3 | 22.4 |
| 3 , 4 | B60 | X | 1325, 69 | X | X | X |
| 6 , 7 | A11 , B7 | X | < | X | X | X |
| 19 , 20 | A2 , B60 | X | 781, 2838 | X | X | X |
| 52 , 53 | A2 | X | 1319, 1594 | X | X | X |
| 54 | B7 | X | < | X | X |  |
| 60 | A2 | X | 219 |  | X | X |
| 87 , 88 | A11 | X | 844, 663 |  |  |  |
| 89 , 90 | B7 , A2 | X | < |  |  |  |
| 91 , 92 | A2 | X | < |  | X |  |
| 108 , 109 | A2 | X | < |  | X | X |

2B.

| C64 | HLA specificity | reactivity over sequential time points  (years post infection) | | | |
| --- | --- | --- | --- | --- | --- |
| peptide | A2, 32  B7, 44 | 17.4 | 19.3 | | 22.4 |
| 5 , 6, 7 | B7 | X | X | < ,< , 117 |  |
| 19 , 20 | A2 |  | X | < , 61 | X |
| 40 | B44 | X | X | < |  |
| 43 , 44 | B44 |  | X | 133, < |  |
| 45 | B7 | X | X | 83 |  |
| 46 | A2 | X | X | 50 |  |
| 52 , 53 | A2 |  | X | 122, 83 | X |
| 54 | B7 |  | X | < |  |
| 74 | B44 |  | X | < | X |
| 77 | B44 |  | X | 78 | X |
| 82 | B7 | X | X | 178 | X |
| 85 , 86 | A2 | X | X | 56, < | X |
| 89 , 90 | A2 , B7 | X | X | 56 | X |
| 92 | A2 | X |  | < |  |
| 108 , 109 | A2 |  | X | < | X |
| 112 | A2 | X | X | < |  |

2C

| C13 | HLA specificity | reactivity over sequential time points  (years post infection) | | | | | |
| --- | --- | --- | --- | --- | --- | --- | --- |
| peptide | A 3, 25  B18, 27 | 10.9 | 12.5 | | 19.2 | 22.8 | |
| 4 , 5 | A3 , B27 | X | X | 1565, 1920 | X | X | 570, 775 |
| 51 | A25 | X | X | 695 |  |  | < |
| 66 | B27 | X | X | 1920 | X | X | 2050 |
| 67 | B27 , A3 |  |  |  |  | X |  |
| 101 | A3 |  | X | 80 |  |  | < |

2D

| C53 | HLA specificity | reactivity over sequential time points  (years post infection) | | | | | |
| --- | --- | --- | --- | --- | --- | --- | --- |
| peptide | A2, 24,  B15, 40 | 12.1 | 17.2 | 18.9 | 21.3 | | 22.6 |
| 3 | B44 | X |  | X |  | < |  |
| 7 | A24 | X |  | X |  | < |  |
| 19 , 20 | A2 | X |  | X | X | 898, 1669 | X |
| 42 , 43 | A2 | X |  | X | X | 1066 | X |
| 46 | A2 | X |  | X | X | < | X |
| 48 , 49 | A2 , B15 | X |  | X | X | < | X |
| 50 | B40 | X |  | X | X | < | X |
| 52 , 53 | A2 , B40 | X | X | X | X | 1139, 1898 | X |
| 60 | A2 | X | X | X | X | 181 | X |
| 61 , 62 | A2 | X | X | X | X | < | X |
| 65 , 66 | A24 | X | X | X | X | 72, 96 | X |
| 67 , 68 | B15 | X |  | X | X | 2048, 3012 | X |
| 69 | A2 , A24 | X | X | X | X | < | X |
| 85 , 86 | A2 |  |  | X | X | < | X |
| 91 , 92 | A2 | X |  |  | X | < | X |
| 108,109 | A2 | X |  |  |  | 54, 72 |  |

2E

| C18 | HLA specificity | reactivity over sequential time points  (years post infection) | | |
| --- | --- | --- | --- | --- |
| peptide | A2, 11  B44, 60 | 10.9 | 11.8 | 12.0 |
| 3 / 4 | B44 / B60 |  | < | X |
| 19 / 20 | B60 / A2 |  | 98, 106 | X |
| 52 / 53 | A2 / B44 |  | < | X |
| 60 / 61 | A2 |  | < | X |
| 62 / 63 | A2 | X | < | X |
| 64 / 65 | B44 / B60 |  | < | X |
| 67 | A11 | X | 76 | X |
| 68 / 69 | A2 | X | 288, 205 | X |
| 74 | B44 |  | < | X |
| 77 | B44 | X | 2144 | X |
| 87 / 88 | A11 | X | 318, 447 | X |
| 90 | A2 | X | < | X |
| 108 / 109 | A2 | X | 68 | X |

2F

| C122 | HLA specificity | reactivity over sequential time points  (years post infection) | | | | | |
| --- | --- | --- | --- | --- | --- | --- | --- |
| peptide | A2, 31  B27, 44 | 15.4 | 17.3 | 20.3 | | 22.2 | 22.8 |
| 19 / 20 | A2 / B44 | X | X | X | 149, 1144 | X | X |
| 38 | A2 | X |  | X | < |  |  |
| 40 | B44 | X | X | X | < | X |  |
| 49 | A2 |  |  | X | < | X | X |
| 62 | A2 | X |  |  | < |  |  |
| 66 / 67 | B27 | X | X | X | 5165 | X | X |
| 69 | A2 | X | X | X | 271 | X | X |
| 74 | B44 | X |  |  | < |  |  |
| 91 / 92 | A2 |  |  | X | < |  | X |
| 108/109 | A2 | X | X | X | 319 | X | X |
